# Evaluation of cardiac fibrosis and subclinical cardiac changes in children with sickle cell disease using magnetic resonance imaging, echocardiography, and serum galectin-3

**DOI:** 10.1007/s00247-023-05750-2

**Published:** 2023-09-16

**Authors:** Reham Wagdy, Alaa Fathy, Abdelaziz Elnekidy, Geylan Salaheldin, Hanan Nazir, Rana Fahmy, Hagar Elkafrawy, Fatma Elkafrawy

**Affiliations:** 1 Department of Pediatrics, Pediatrics Cardiology Unit, Faculty of Medicine, Alexandria University, Alexandria, 21648 Egypt; 2 Department of Radiology, Faculty of Medicine, Alexandria University, Alexandria, Egypt; 3 Department of Pediatrics, Faculty of Medicine, Hematology Unit, Alexandria University, Alexandria, Egypt; 4 Department of Medial Biochemistry, Faculty of Medicine, Alexandria University, Alexandria, Egypt

**Keywords:** Cardiac fibrosis, Children, Galectin-3, Myocardial fibrosis, Myocardial performance index, Sickle cell disease, Tissue Doppler echocardiography

## Abstract

**Background:**

Myocardial fibrosis has recently been proposed as one of the contributing factors to the diverse pathogenicity of cardiomyopathy in sickle cell disease.

**Objective:**

In this study, cardiac fibrosis and subclinical cardiac changes in children with sickle cell disease were evaluated using cardiac magnetic resonance imaging (MRI), tissue Doppler echocardiography and serum galectin-3.

**Materials and methods:**

The study included 34 children with sickle cell disease who were compared with a similar number of healthy controls. Cardiac MRI was used to evaluate late gadolinium enhancement, native T1 mapping, extracellular volume, and T2* for estimation of iron load. Cardiac function and myocardial performance index (MPI, evaluated by tissue Doppler echocardiography) and serum galectin-3 were compared to controls.

**Results:**

The mean age of the included patients was 13.3 ± 3.2 years. Myocardial iron load by T2* was normal. The mean level of extracellular volume (35.41 ± 5.02%) was significantly associated with the frequency of vaso-occlusive crises (*P* = 0.017) and negatively correlated with hemoglobin levels (*P* = 0.005). Galectin-3 levels were significantly higher among cases than controls (*P* = *0.00)*, at a cutoff value on the receiver operating characteristic curve of 6.5 ng/ml, sensitivity of 82.5% and specificity of 72.8%. The extracellular volume was significantly higher in cases, with a MPI > 0.4.

**Conclusion:**

Diffuse interstitial myocardial fibrosis can be detected early in children with sickle cell disease using T1 mapping and is associated with a high frequency of vaso-occlusive crisis. MPI of the left ventricle and serum galectin-3 are recommended screening tools for subclinical cardiac abnormalities.

**Graphical abstract:**

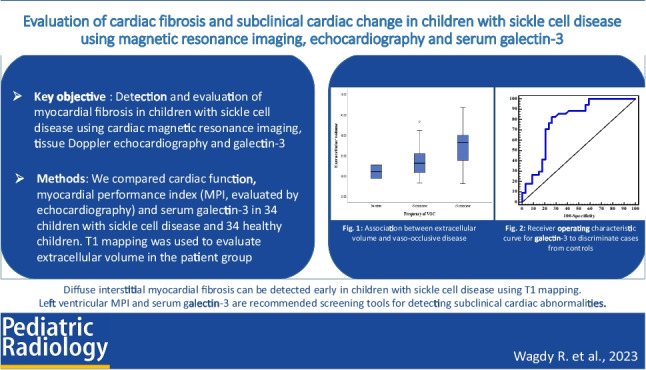

**Supplementary information:**

Supplementary material is available at 10.1007/s00247-023-05750-2.

## Introduction

Sickle cell disease is the most common hereditary hemolytic anemia in the United States of America and one of the prevalent hereditary hemolytic anemias in sub-Saharan Africa, the Mediterranean region and central India [1]. Cardiovascular complications are a notable cause of premature deaths in adults with sickle cell disease, and are responsible for approximately 32% of deaths of individuals in their mid-thirties to mid-fifties [1, 2]. In children, the disease profile has been changed, mostly due to screening programs, vaccine therapy and antibiotic prophylaxis. Consequently, acute severe infections are no longer the main cause of death in children [3].

The predominant manifestations of the disease are related to vaso-occlusive crises. Repeated episodes of tissue ischemic reperfusion injury, in addition to the effect of hemolysis, result in a chronic vasculopathy with an inflammatory state, which leads to chronic end-organ damage [1, 3]. Multiple pathological mechanisms might contribute to sickle cell cardiomyopathy, including chronic anemia, recurrent vaso-occlusive crises and iron overload with excess production of reactive oxygen species, in addition to pulmonary, renal and hepatic damage [4–6].

Recent studies have reported cardiac fibrosis in adult patients with sickle cell disease as a novel mechanism for cardiac dysfunction [7]. Cardiac fibrosis is a complex heterogeneous process that is characterized by excessive accumulation of extracellular matrix in response to pathological stimuli [8]. Cardiac fibrosis may be replacement fibrosis, which develops in response to myocardial infarction, or reactive fibrosis which develops in response to pressure and volume overload [9, 10].

The noninvasive detection of myocardial fibrosis is challenging. Cardiac magnetic resonance imaging (MRI) studies using late gadolinium enhancement are currently the gold standard noninvasive method to detect focal myocardial fibrosis. However, focal myocardial fibrosis has only been reported in a few patients with sickle cell disease [4, 11–13]. Late gadolinium enhancement is insensitive to diffuse myocardial fibrosis [13, 14]. T1 mapping is a new technique that has emerged to overcome the limitations of late gadolinium enhancement in detecting diffuse myocardial fibrosis by quantifying the expansion of the extracellular matrix [15]. Cardiac MRI measurement of myocardial T1 relaxation times before and after gadolinium administration has been used to quantify the myocardial extracellular volume [14].

Other noninvasive imaging modalities have been employed to detect myocardial fibrosis including tissue Doppler echocardiography, nuclear imaging and cardiac computed tomography [16–18]. In addition, some laboratory biomarkers can aid in both detection and risk stratification of cardiac fibrosis [19]. Galectin-3 has been suggested to be a marker for cardiac inflammation and fibrosis as it is readily expressed on the cell surface and secreted by the injured inflammatory cells [20, 21].

This study evaluated cardiac fibrosis in children with sickle cell disease using cardiac MRI and also aimed to detect the effectiveness of tissue Doppler echocardiography and serum galectin-3 in identifying subclinical cardiac abnormalities indicative of myocardial fibrosis.

## Patients and methods

### Study design and population

This prospective controlled study was approved by the ethical committee of Alexandria Faculty of Medicine according to the Declaration of Helsinki (IRB: 0201617). Informed consent was obtained from parents of all children enrolled in the study. A total of 68 children participated in this study between September 2021 and June 2022. The enrolled children included two groups, 34 children in each. The patient group included children between 8 and 18 years of age who were diagnosed with sickle cell disease by hemoglobin electrophoresis and recruited consecutively from the Hematology Clinic of the Pediatric Department of our institute. The second (control) group included healthy children (no chronic medical diseases) matched for age and sex with the patient group. Patients with overt heart failure and those with severe renal impairment (glomerular filtration rate <30 mL/min/1.73 m^2^) which is a contraindication to contrast administration were excluded from the study. Enrolment of children with recent vaso-occlusive  crises was delayed by two weeks aiming to perform cardiac MRI under more convenient circumstances on a cooperative child. This precaution was taken as most of these crises were moderate to severe painful crises that required hospitalization of critically ill, irritable children. This delay was also important because some authors [22] have reported elevated galectin-3 level during the first 48 h of a vaso-occlusive crisis, which might have confounded our results.

According to the study by Niss et al. [7], a minimum sample size of 15 children per group is required to compare functional and structural cardiac parameters between patients (children with sickle cell disease) and control children using two-sample t test power analysis, which has 80% power and a target significance level of 5%. The sample size was calculated using the NCSS 2004 and PASS 2000 programs  (http://www.ncss.com).

### Methods

The demographic data and clinical profile of the patients including the frequency of vaso-occlusive crises, sites of pain, blood transfusion and hydroxyurea treatment were obtained. Cases and control subjects underwent routine laboratory and imaging investigations described in the following sections.

#### Serum galectin-3

Blood samples from both patients and control subjects were collected for the measurement of galectin-3 levels. Samples were coagulated at room temperature for 15 min, then centrifuged at 3,000rpm for 10 min. Serum was collected and stored at − 80^o^C until the time of assay. Galectin-3 was measured using enzyme-linked immunosorbent assay (ELISA) kits (Human Galectin-3® assay; Cat# BYEK2857 Chongqing, China), according to the manufacturer’s recommendations [23].

#### Echocardiography

Echocardiographic assessment was performed using a Philips HD 11 ultrasound echocardiography machine (Bothell, WA) with a transducer of 2–4 MHz at the Pediatric Cardiology Clinic of our institute. All echocardiographic measurements were performed in three heart cycles and the mean value was used for analysis. Echocardiographic studies were performed by a single pediatric cardiologist (R.W.) with 15 years of experience. Echocardiographic measurements were performed using 2-dimensional, M-mode Doppler echocardiography and tissue Doppler imaging, because it is a reproducible tool for the detection of myocardial fibrosis [16–18, 24]. The estimated variables of left ventricular function were ejection fraction and peak early (E) and late (A) diastolic inflow velocities of the left ventricle [25]. Tissue Doppler echocardiography was performed with the sample volume positioned at the lateral and medial corners of the mitral valve at the basal left ventricular wall to obtain the following velocities: peak systolic (Sm and Ss), peak early (Em and Es) and peak late (Am & As) diastolic annular velocities with Em/Am and Es/As ratio. The global myocardial performance index (MPI or Tei index, the sum of isovolumic contraction times and isovolumic relaxation times divided by ejection time) was calculated for the septal and lateral leaflets of the mitral valve [25, 26]. The mean normal MPI value of the left ventricle is 0.39 ± 0.05 or less [27].

#### Cardiac magnetic resonance imaging

Cardiac MRI was conducted on a 1.5 tesla MRI scanner (Philips Ingenia MR Systems, Eindhoven, Netherlands). All images were electrocardiograph (ECG) gated using retrospective gating, with 25 images reconstructed per cardiac cycle. The estimated volume and function cardiac MRI measurements were independently performed by two radiologists (F. E. with 5 years of experience and G.S. with 3 years of experience) blinded to the case/control status of each scan and the results were compared to the values reported individually by van der Ven et al. and Robbers-Visser et al. for healthy children matched by age and sex [28, 29]. The T2* sequence for measuring the myocardial T2* value, myocardial iron concentration (MIC) and liver iron concentration (LIC) were calculated and iron overload risk stratification in patients was performed according to Abdallah et al. [30] and others [31, 32] (Supplementary Material 1).

#### T1 mapping

The motion-corrected (MOCO) modified Look-Locker Inversion recovery (MOLLI) sequence was acquired before and 10 min after bolus contrast administration with variable inversion preparation times. The myocardial extracellular volume was assessed using the equation reported previously [33]. Our calculated extracellular volume was compared to the cutoff value of 20.8 ± 2.4% reported by Pagano et al., who collected T1 mapping data from 48 healthy pediatric patients (age: 14 ± 3 year) [34]. For ethical considerations, contrast was not administered to healthy volunteers. Therefore, extracellular volume values were calculated only for children with sickle cell disease and not for the control group, which is similar to other studies [35, 36]. Late gadolinium enhancement was used for detection of focal myocardial hyperenhancement (focal myocardial fibrosis) against normal nulled myocardium. All details are available in Supplementary Material 1 [34–36]

The patient group was further subdivided into subgroup I: < median age and subgroup II: > median age of the patient group and the galectin-3 levels, MPI and extracellular volumes of both subgroups were compared.

### Statistical analysis

Data were analyzed using IBM SPSS software package version 20.0. (Armonk, NY: IBM Corp). Student *t*-test was used to compare two groups for normally distributed quantitative variables, The Pearson coefficient r, Pearson correlation, and Spearman correlation, r_s_ were used to correlate two normally distributed quantitative variables and the Mann‒Whitney test was used. Our results were considered statistically significant at *P*-value ≤ 0.05. The significance of the receiver operating characteristic (ROC) curve was judged at the 5% level. The area under the ROC curve denotes the diagnostic performance of the test. An area of more than 50% gives acceptable performance and an area of approximately 100% is the best possible performance for the test.

## Results

### Characteristics of study population

**Fig. 1:**
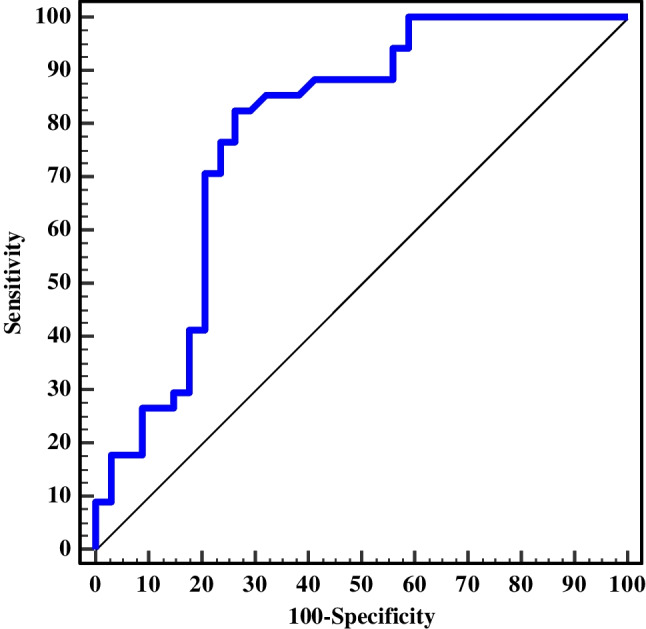
Receiver operating characteristic (ROC) curve for serum galectin-3 to discriminate patients with sickle cell disease (*n*=34) from control subjects (*n*=34). The area under the ROC curve was 0.786 (*P*<0.001) with a 95% confidence interval of 0.674–0.899. The cut-off value for serum galectin-3 was 6.555 ng/ml (chosen according to the Youden index) at which sensitivity=82.4%, specificity=73.5%, positive predictive value=75.7% and negative predictive value = 80.6%

The study population included a patient group of 34 children with sickle cell disease, of whom 19 were males (mean age of 13.3 ± 3.2 years) and a control group consisting of a similar number of age- and sex-matched healthy children. The consecutive selection of the patients from the hematology clinic was based on random chance. We excluded ten potential patients for the following reasons: five had experienced a recent vaso-occlusive crisis and (as per the study protocol), were advised to return in two weeks but did not; three had severe claustrophobia; and two patients had severe renal impairment (GFR < 30 mL/min/1.73 m^2^). The demographic and laboratory data of the included children are summarized in Table 1. Almost all patients (93.4%) had a history of recurrent vaso-occlusive crises; 50% having had more than three attacks per year, 88.2% were on hydroxyurea treatment. Back pain and abdominal pain were equally prevalent (68%) symptoms. The least prevalent site of pain was the chest (2%). Only 55.9% of patients had a history of previous blood transfusion. The mean serum galectin-3 level was significantly higher among patients than among control subjects (7.75 ± 1.86 ng/ml versus 6.04 ± 1.64 ng/ml, respectively, *P* < 0.001). Based on the ROC curve, the best cutoff value of galectin-3 for differentiating between patients and control subjects was 6.6 ng/ml, with a sensitivity of 82.5% and specificity of 72.8% (Fig. 1).

**Table 1:** Demographic characteristics of and laboratory tests in the study groups

	Cases (*n*=34)	Controls (*n*=34)	*P-*value
Sex *n* (%):			0.894
Male	19 (55.9)	19 (54.3)	
Female	15 (44.1)	16 (45.7)	
Age (years)			0.068
Mean ± SD	13.3 ± 3.2	12.0 ± 2.4	
Median (min.–max.)	13.5 (8.0–18.0)	9.0 (9.7–16.7)	
Weight (kg)			0.156
Mean ± SD	39.7 ± 16.2	35.3 ± 8.1	
Median (min.–max.)	36.5 (19.0–96.0)	35.0 (19.0–70.0)	
Height (cm)			0.099
Mean ± SD	141.5 ± 16.9	136.0 ± 9.2	
Median (min.–max.)	143.5 (101.0–169.0)	138.0 (115.0–170.0)	
Hemoglobin gm/dl			** < 0.001**
Mean ± SD	9.38 ± 1.75	11.57 ± 0.66	
Median (Min.–Max.)	8.9 (6.5–13.1)	11.5 (10.5–13.0)	
Galectin 3 ng/dl			** < 0.001**
Mean ± SD	7.75 ± 1.86	6.04 ± 1.64	
Median (min.–max.)	7.41 (5.80–16.85)	6.13 (1.47–9.15)	
Ferritin last 4 years mg/dl			-
Mean ± SD	1367.62 ± 2259.74	-	
Median (min.–max.)	500.0 (12.0–10262.0)	-	

*Max* maximum, *Min* minimum, *SD* standard deviation

Bold indicates statistical significance (*P* ≤ 0.05)

### Echocardiography

Although conventional echocardiography revealed that all patients had normal systolic function, the mean ejection fraction of patients was significantly lower than that of the control group (61.16 ± 6.09% versus 66.43 ± 5.98%, *P*=0.001). However, tissue Doppler variables of the systolic and diastolic function at the lateral mitral annulus and the systolic variables of the medial mitral annulus were significantly different for cases when compared to controls (Table 2). Additionally, the MPI of the lateral and medial (septal) mitral leaflets were significantly higher in patients than in controls. Of the 25 patients (73.2%) with a high (> 0.4) left ventricular MPI, 20 showed severe impairment of left ventricular function (MPI ≥ 0.6). Restrictive diastolic impairment (E/A>2) was identified in 15 (44%) patients.

**Table 2:** Echocardiographic assessment of the study groups

	Patients (*n*=34)	Control group (*n* =34)	*P-*value
I-Conventional echocardiography of LV			
EF (%)			**0.001**
Mean ± SD	61.16 ± 6.09	66.43 ± 5.98	
Median (min.–max.)	62.0 (52.5–75.0)	66.0 (56.0–78.0)	
FS (%)			0.158
Mean ± SD	32.92 ± 4.7	34.46 ± 4.03	
Median (min.–max.)	33.3 (26.9–44.1)	35.0 (28.0–44.0)	
E/A ratio			0.189
Mean ± SD	1.86 ± 0.43	1.71 ± 0.18	
Median (min.–max.)	2.0 (1.0–2.6)	1.72 (1.3–2.0)	
TAPSI cm/s			** < 0.001**
Mean ± SD	1.65 ± 0.31	2.09 ± 0.36	
Median (min.–max.)	1.6 (1.1–2.5)	2.25 (1.48–2.71)	
II-Pulsed tissue Doppler of mitral annuls leaflets (septal & lateral)			
Ss (cm/s)			** < 0.001**
Mean ± SD	9 ± 2	16 ± 2	
Median (min.–max.)	9 (6–14)	16 (12–18)	
Es (cm/s)			0.844
Mean ± SD	16 ± 3	16 ± 2	
Median (min.–max.)	16 (13–23)	16 (11–18)	
As (cm/s)			** < 0.001**
Mean ± SD	7 ± 3	12 ± 2	
Median (min.–max.)	5 (4–15)	11 (8–15)	
Es/As			** < 0.001**
Mean ± SD	2.73 ± 0.79	1.36 ± 0.17	
Median (min.–max.)	2.8 (0.85–3.9)	1.37 (1.1–1.7)	
MPIs			** < 0.001**
Mean ± SD	0.81 ± 0.28	0.33 ± 0.11	
Median (min.–max.)	0.8 (0.42–1.8)	0.3 (0.21–0.37)	
Sm (cm/s)			** < 0.001**
Mean ± SD	0.08 ± 0.02	0.15 ± 0.02	
Median (min.–max.)	0.08 (0.05–0.13)	0.15 (0.11–0.18)	
Em (cm/s)			** < 0.001**
Mean ± SD	17 ± 3	15 ± 2	
Median (Min.–Max.)	17 (1–24)	16 (11–18)	
Am (cm/s)			** < 0.001**
Mean ± SD	8 ± 0.04	11 ± 2	
Median (min.–max.)	7 (4 – 16)	11 (9–14)	
Em/Am			** < 0.001**
Mean ± SD	2.65 ± 1.12	1.32 ± 0.13	
Median (min.–max.)	2.5 (0.8–4.5)	1.3 (1.04–1.6)	
MPIm	0.69 ± 0.59	0.36 ± 0.57	** < 0.001**
Mean ± SD			
Median (min.–max.)	0.5 (0.17–2.0)	0. 3 (0.25–0.43)	

*Am* peak late diastolic velocity at lateral mitral valve annulus, *As* peak late diastolic velocity at septal mitral valve annulus, *E/A* peak earl diastolic velocity of mitral flow/peak late diastolic velocity of mitral flow, *EF* ejection fraction, *Em* peak early diastolic velocity at lateral mitral valve annulus, *Es* peak early diastolic velocity at septal mitral valve annulus, *FS* fractional shortening, *MPIm*, myocardial performance index at lateral leaflets, *MPIs* myocardial performance index at septal leaflets, *SD* standard deviation, *Sm* peak velocity of systolic excursion of lateral mitral, *Ss* systolic excursion of septal mitral, *TAPSI* tricuspid annular plane systolic excursion tricuspid valves. Bold indicates statistical significance (*P*≤0.05)

### Cardiac magnetic resonance imaging

**Fig. 2:**
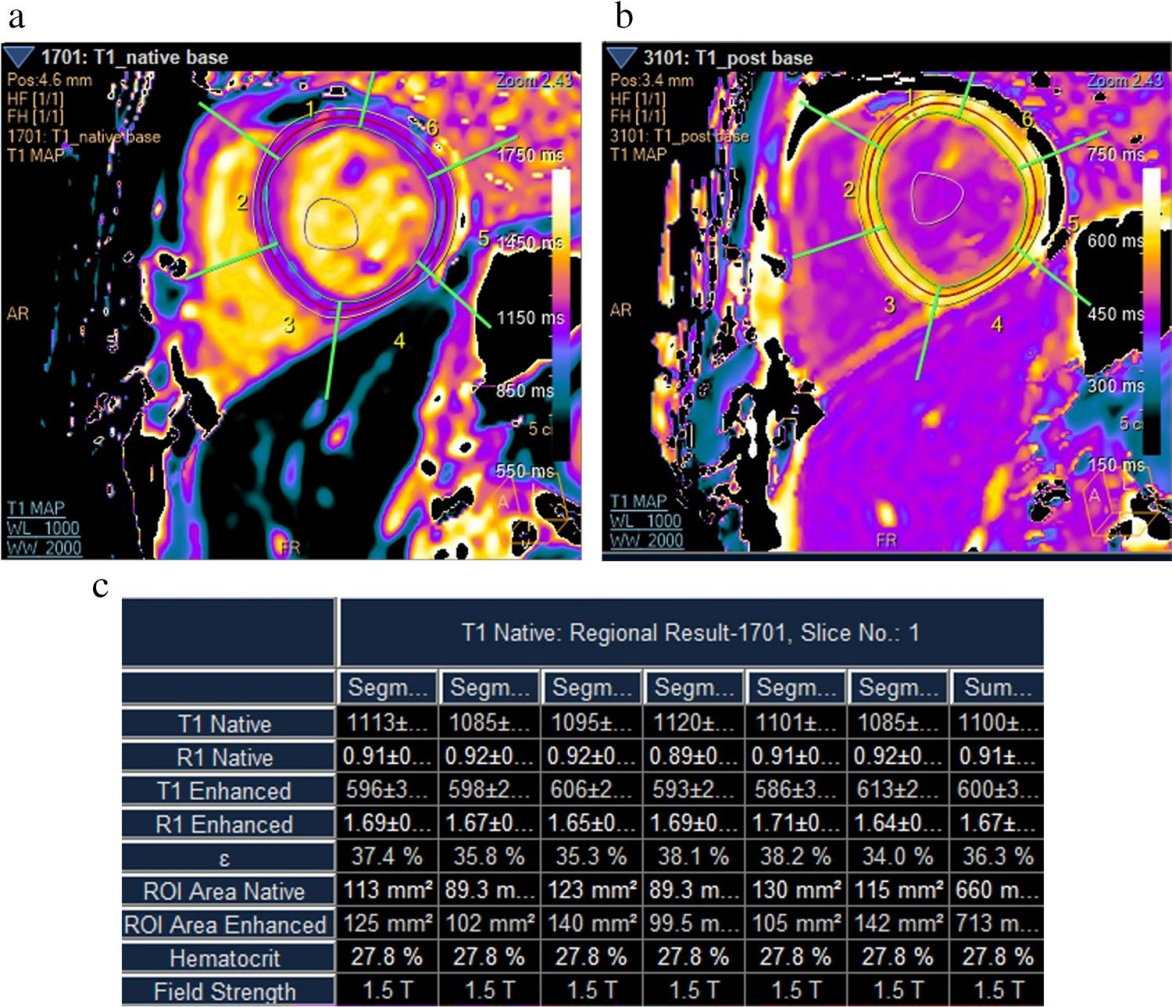
Left ventricular (LV) basal cut short axis contrast enhanced cardiac magnetic resonance T1 mapping sequence in a 15-year-old boy with sickle cell disease pre- (**a**) and postcontrast (**b**) T1 color-coded maps. **c **Table shows the generated results for native T1, enhanced T1 and extracellular volume, with high extracellular volume in all LV basal segments and a mean of 36.3%

**Fig. 3:**
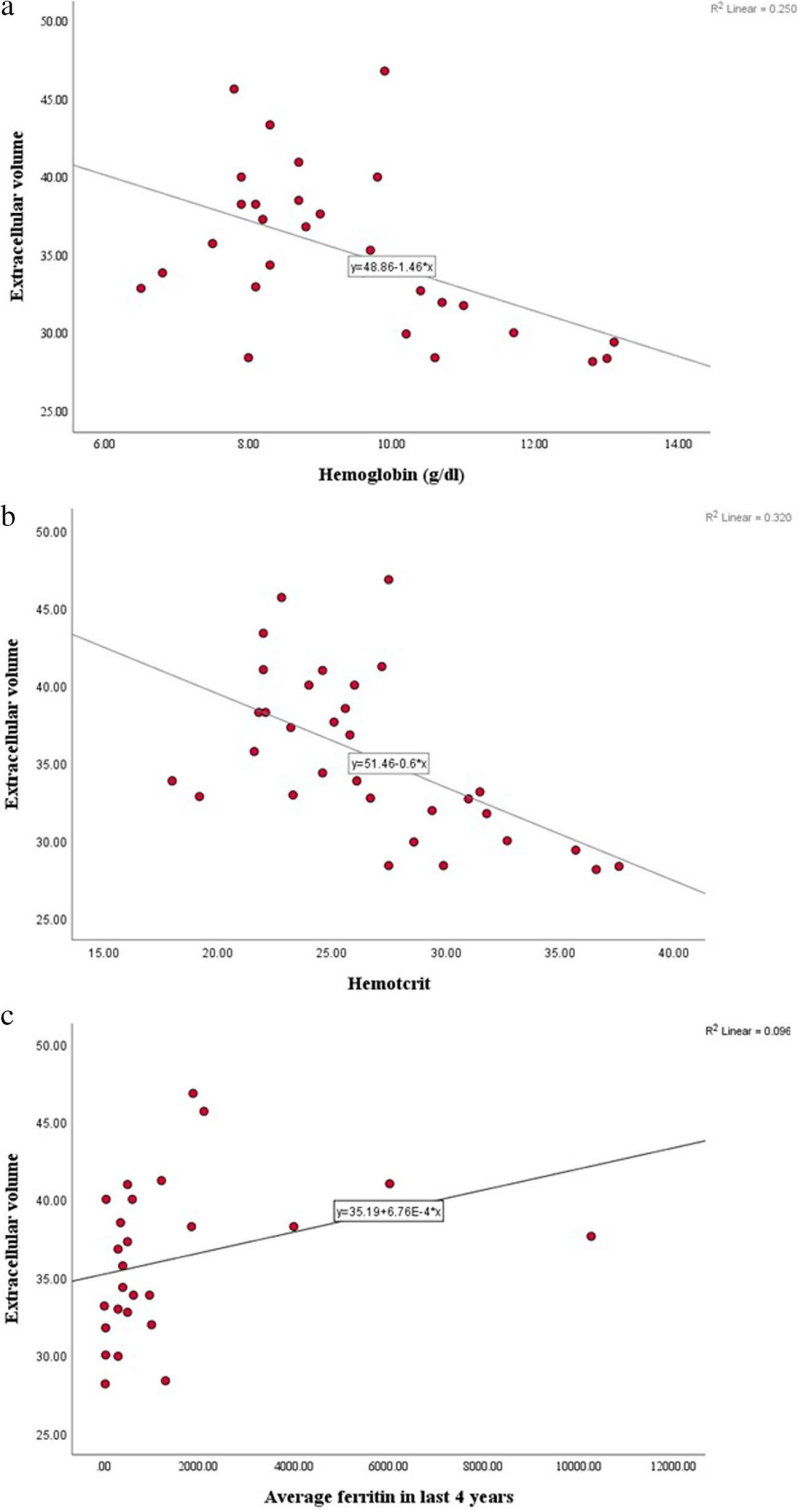
Correlation between extracellular volume and laboratory risk factors. **a** Significant  (*P*<0.05) negative correlation between average extracellular volume and hemoglobin (*P* = 0.005). **b** Significant negative correlation between average extracellular volume and hematocrit (*P* < 0.001*). **c** Significant positive correlation between average extracellular volume and average ferritin in the preceding four years (*P* = 0.006). *Z* Wilcoxon test, *r*_*s*_ Spearman correlation, *SD* standard deviation

**Fig. 4:**
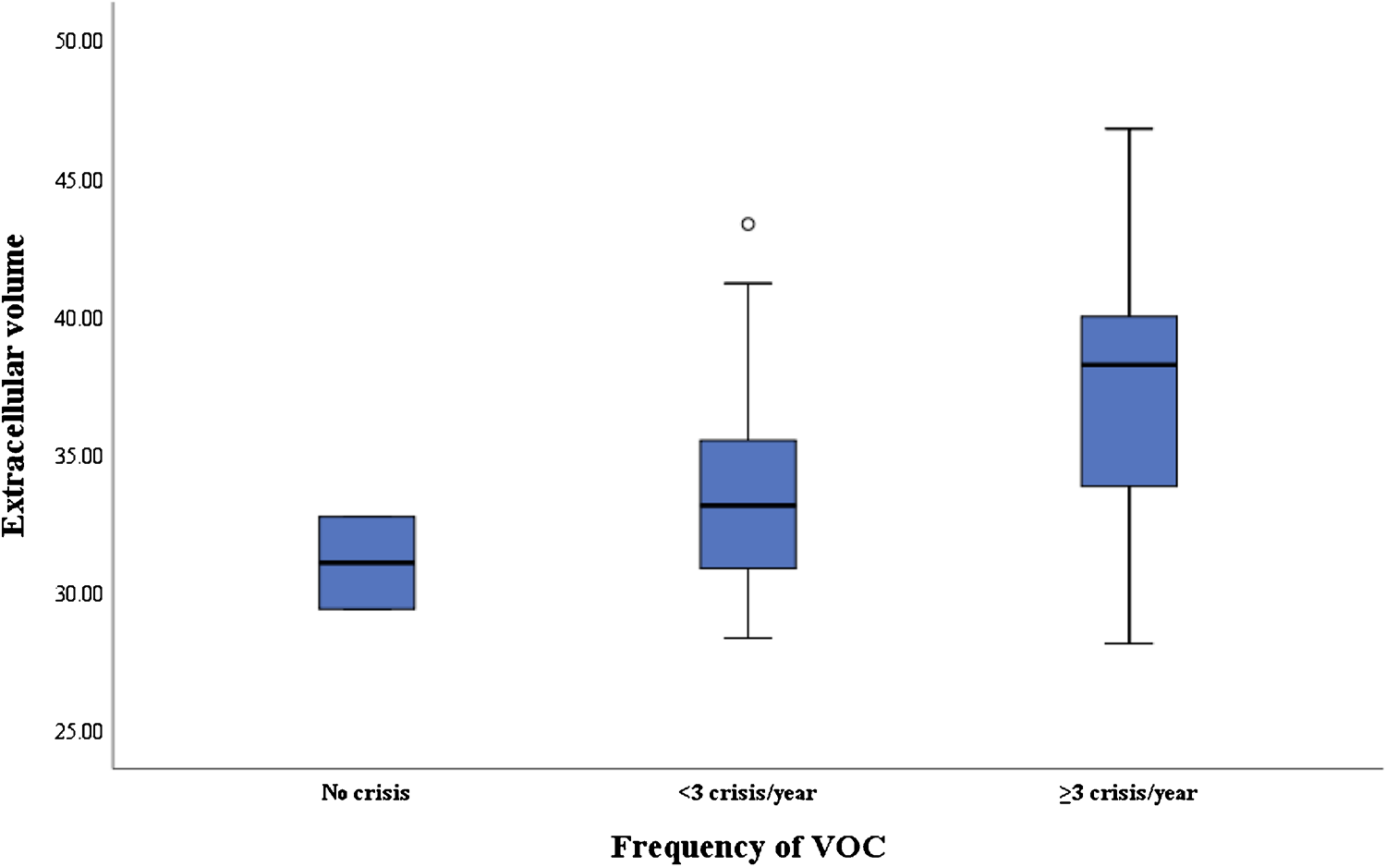
Association between frequency of vaso-occlusive crises (VOC) and extracellular volume in children with sickle cell disease

The mean indexed left ventricular end-systolic volume (35.54 ± 9.44 ml/m^2^) and systolic function (ejection fraction: 65.29 ± 5.8%) estimated by cardiac MRI were within the normal range for age and sex in all patients. The mean indexed left ventricular end-diastolic volume (LV-EDVI: 102.29 ± 19.8ml/m^2^) and the indexed left ventricular mass (74.62 ± 15.37gm/m^2^) were increased for age and sex in all patients. The left ventricular ejection fraction on cardiac MRI was positively correlated with the ejection fraction estimated by echocardiography and with the velocity of lateral and medial mitral leaflets (*P* < 0.008 and *P* < 0.001, respectively). None of the patients was found to have myocardial iron overload. The mean myocardial iron T2* was 33.58 ± 6.83ms and the mean MIC was 0.68 ± 0.19mg/g. The mean hepatic iron T2* was 14.64 ± 9.66ms and the mean LIC of the patients was 4.97 ± 7.67mg/g. We found that 20 patients (59%) had normal hepatic iron concentrations, three patients (8.8%) had moderate hepatic iron overload, four (11.8%) had severe hepatic iron overload, while the remaining seven (20.6%) had mild hepatic iron overload. Of the control group, 15 had non-contrast cardiac MRI and the native T1 (precontrast) was estimated. The mean native T1 was significantly higher for patients when compared to control subjects (1,119.28 ± 54.38ms versus 1,070.77 ± 66.77ms, *P*=0.014). No significant difference was found between the septal and lateral wall extracellular volumes, (35.84 ± 4.81% versus 34.55 ± 4.71%, *P* 0.069*)*. The average extracellular volume values (the average of all myocardial segments) was used. We found that the average extracellular volume was significantly increased in 100% of patients when compared to the normal cutoff for age and sex (35.41 ± 5.02% versus 20.8 ± 2.4%). Figure 2 shows a high extracellular volume level for a 15-year-old boy with sickle cell disease. A significant negative correlation was found between the extracellular volume and hemoglobin and hematocrit percentage (*r*=− 0.5, *P*=0.005 and *r*=− 0.566, *P*< 0.00, respectively) as illustrated in Fig. 3, which also illustrates a positive correlation between ferritin in the preceding four years (*r*=0.62, *P*=0.006). A significant association between a high extracellular volume and the frequency of vaso-occlusive crises (*t*=− 2.536, *P*=0.017) was noted (Fig. 4). Stratification of the children with sickle cell disease according to number of blood transfusions per year revealed a statistically significant difference in the mean extracellular volume between the subgroups, being highest for transfusion-dependent (TD) patients (non-TD: 34.2 ± 4.6%, TD 1–3 times per year: 33.53 ± 4.09%, TD 4–6 times per year: 38.56 ± 3.95% and TD > 6 times per year: 39.17 ± 2.48%). Children with sickle cell disease were divided according to age into two subgroups: subgroup I (age < 13.5 years) and subgroup II (> 13.5 years); galectin-3 levels were significantly higher in the older group (*P* = 0.01)*.* Although the mean MPIm and extracellular volume were higher for the older group, they did not reach statistical significance (Table 3). Additionally, the extracellular volume was positively correlated with restrictive diastolic dysfunction. The extracellular volume values were significantly higher among children with sickle cell disease with E/A > 2 compared to those with E/A < 2 (38.02 ± 5.37% versus 33.8 ± 4.14%, *P* = 0.03), as shown in Table 4.

**Table 3:** Effect of age on extracellular volume, galectin-3 and myocardial performance index

	Sickle cell patients		*P-*value^a^
	Subgroup I ≤ 13.5 years (*n*=21)	Subgroup II > 13.5 years (*n*=13)	
Extracellular volume %			0.786
Mean ± SD	35.23 ± 4.68	35.72 ± 5.72	
Median (min–max)	34.35 (28.15–46.8)	35.73 (28.35–45.65)	
MPIm			0.458
Mean ± SD	0.6 ± 0.51	0.81 ± 0.7	
Median (min–max)	0.51 (0.17–2.0)	0.51 (0.17–2.0)	
Galectin-3			**0.017**
Mean ± SD	7.23 ± 0.98	8.6 ± 2.59	
Median (min–max)	7.3 (5.8–9.3)	7.67 (6.79–16.845)	

*MPIm* myocardial performance index at the lateral leaflets of the mitral valve, *SD* standard deviation

Bold indicates statistical significance (*P* ≤ 0.05)

**Table 4:** Relation between extracellular volume and peak diastolic velocities

	Diastolic function of left ventricle		*P*-value
	E/A < 2 (*n* = 19)	E/A > 2 (*n* = 15)	
Extracellular volume %			**0.035**
Mean ± SD	33.8 ± 4.14	38.02 ± 5.37	
Median (min–max)	33.85 (28.15–40.95)	38.5 (31.75–46.8)	

*A* peak late diastolic inflow velocity, *E* peak early diastolic inflow velocity, *Max* maximum, *Min* minimum, *SD* standard deviation

Bold indicates statistical significance (*P*≤0.05)

A positive correlation between left ventricular MPIm and extracellular volume was found. Patients with MPIm > 0.4 demonstrated significantly higher values of extracellular volume than those with left ventricular MPIm < 0.4, as illustrated in Table 5.

**Table 5:** Relation between myocardial performance index, extracellular volume and galectin-3

	MPIm		*P*-value
	Abnormal (> 0.4) (No = 24)	Normal (≤ 0.4) (No = 10)	
Extracellular volume %			**0.03**
Mean ± SD	38.17 ± 4.25	34.43 ± 5.02	
Median (min–max)	39.32 (31.75–45.65)	33.05 (28.15–46.8)	
Galectin-3 ng/ml			0.705
Mean ± SD	8.34 ± 3.12	7.51 ± 0.97	
Median (min–max)	7.81 (5.835–16.845)	7.4 (5.80–9.48)	

*MPIm* myocardial performance index at lateral leaflets of mitral valve, *SD* standard deviation

Bold indicates statistical significance (*P* ≤ 0.05)

## Discussion

In the current study, diffuse myocardial fibrosis was found to be a common subclinical feature in children with sickle cell disease. Using late gadolinium enhancement, increased extracellular volume was found in all patients, even in the absence of focal fibrosis. Our results highlight an important and underrecognized mechanism of heart pathology in sickle cell disease that appears to predate the development of cardiac dysfunction. A few studies have reported this abnormality in adult patients and this phenomenon has not been fully studied in children [7]. Niss et al. used T1 mapping to measure extracellular volume in a cohort study that included 25 adult patients with sickle cell disease. They reported markedly increased extracellular volume in all patients when compared to a control group (mean 44% ± 8% versus 26 ± 2%, *P* < 0.0001), indicating diffuse myocardial fibrosis [13]. Our results are consistent with this, as we found that the mean extracellular volume among children with sickle cell disease was 35.41 ± 5.02%. This value is higher than most previously published extracellular volume values in other fibrotic heart diseases, such as hypertrophic cardiomyopathy [37, 38]. Similarly, Morin et al. conducted a study that included 26 adolescents with sickle cell disease with a median age of 17.5 years; 96% of them had increased extracellular volume (median 30.5%) despite the absence of focal myocardial fibrosis by late gadolinium enhancement [39]. Native T1 values in the present study were significantly increased in children with sickle cell disease compared to control group, which matches with the findings reported by Niss et al. (patients, 1,008 ± 67ms vs control subjects, 942 ± 25ms; *P* < 0.001) [7]. The role of anemia in the development of myocardial fibrosis in sickle cell disease is unknown. However, a negative correlation between the extracellular volume and hemoglobin level was observed (*r* =  − 0.5, *P* = 0.005), which is in agreement with the findings of Niss et al. [13]. Despite high extracellular volume being detected in all of our patients, none had late gadolinium enhancement, which suggests that patients with sickle cell disease may have myocardial fibrosis and ischemia in the absence of major coronary artery disease. In an adult study, Bratis et al. [40] postulated that repeated vaso-occlusive crises resulted in a reduced vascular bed by impairment of vasoreactivity and reperfusion-induced vascular injury at the microvascular level. In agreement with this hypothesis, extracellular volume was significantly associated with the frequency of vaso-occlusive crises in our patients (*t* =  − 2.536, *P* = 0.017)*.* Although blood ferritin levels were high in all patients, the T2* values were normal, which minimized the possibility that cardiac siderosis was the underlying cause of the myocardial alterations in children with sickle cell disease. This is in line with the findings of Junqueira et al. [41] who reported that patients with sickle cell disease rarely experience cardiac siderosis. This value was normal in our patients. However, theoretically, if there is myocardial iron overload, this will result in a decrease, “not increase,” in native T1 values, as reported by Sado et al. [42]. In the present study, the extracellular volume was highest among TD patients who required more than six transfusions per year, and lowest for non-TD patients or those who required fewer than three transfusions per year. In the present study, the extracellular volume was significantly associated with the frequency of vaso-occlusive crises, supporting the ischemic-reperfusion hypothesis.

We were interested in evaluating left ventricular function among the patient group using echocardiography as myocardial fibrosis might affect cardiac function at a subclinical level. Combined systolic and diastolic function of the left ventricle was evaluated using the MPI, which was significantly higher in the children with sickle cell disease than in the control group. However, all patients showed normal systolic function on conventional echocardiography. Similarly, Caldas et al. [43] studied 107 children with sickle cell disease (mean age 10.1 ± 4.7years) and reported higher MPI in the left ventricle for patients than for the control group (*P* = 0.00). Other parameters of systolic and diastolic function of the left ventricle on tissue Doppler echocardiography were significantly decreased in patients compared to healthy control children. Eddine et al. [44] conducted a study of 55 children and young adults with sickle cell disease (6–21 years old) and reported similar results. Ghaderian et al. [45] conducted a study of 64 Iranian children with sickle cell disease (mean age 11.7 ± 5.5 years) compared to 50 healthy control subjects and reported no significant differences in tissue Doppler echocardiographic systolic, diastolic or MPI of the left ventricle. Surprisingly, some studies have reported no significant difference in echocardiographic variables between adult patients and control subjects, e.g., Dabirian et al. conducted a study of 30 asymptomatic patients ages 18–40 years. Age is not the only risk factor for myocardial injury, there are many other confounding factors, such as vaso-occlusive crises, anemia, transfusion dependence and even variations in genotype–phenotype patterns [45–47]. Interestingly, 44% of the children with sickle cell disease in the current study showed impaired left ventricular relaxation with a restrictive pattern (E/A > 2), which was positively correlated with high values of extracellular volume versus the subgroup with E/A < 2 (36.12 ± 5.08% versus 32.1 ± 3.86%, *P* = 0.038). This clarifies the impact of interstitial myocardial fibrosis on diastolic dysfunction and the restrictive pattern of sickle cell disease cardiomyopathy early in childhood. Similar findings were reported by Niss et al. [15] and Alsaied et al. [48] in adults with sickle cell disease.

The current study has demonstrated a correlation between the MPI and diffuse cardiac fibrosis, which has not previously been reported. Additionally, increased extracellular volume was correlated with a higher MPI. The mean extracellular volume was significantly higher for the subgroup with MPI > 0.4 (impaired) than the subgroup with MPI < 0.4 (38.1 ± 4.25% versus 34.43 ± 5.02%, respectively, *P* = 0.03).

Galectin-3, a novel biomarker, has been predicted to play an important regulatory role in cardiac fibrosis and remodeling. In fact, galectin-3 expression appears to begin prior to the onset of heart failure [49, 50]. There are limited data on serum galectin-3 levels in children and most studies have been conducted on congenital heart diseases. Kotby et al. [51] studied galectin-3 among children with heart failure and found increased serum galectin-3 in patients compared to control subjects (*P* > 0.001) at a galectin-3 cutoff value of 3.5 ng/ml. In the current study, galectin-3 levels were significantly higher among children with sickle cell disease than among healthy control subjects (*P* < 0.001). At a cutoff value for galectin-3 of 6.5 ng/ml, the ROC curve showed a sensitivity of 82.5% and a specificity of 72.8%, and patients were discriminated from control subjects. These finding are in agreement with the results of Wagdy et al. [5], who reported a similar finding among Saudi Arabian children with sickle cell disease at a cut off of 5.1 ng/ml. To our knowledge, no other studies have investigated galectin-3 levels in chronic hemolytic anemia. Of note, the patient subgroup with left ventricular MPI > 0.4 exhibited higher galectin-3 levels than the subgroup with left ventricular MPI < 0.4. However, this difference did not reach statistical significance and needs further study. Finally, it was interesting to assess the effect of the chronicity of sickle cell disease on the heart through subgrouping the patients based on median age. A significantly higher level of galectin-3 was found in subgroup II (age> 13.5 years) than in subgroup I (age< 13.5 years). Higher extracellular volume and MPI were also found in the older subgroup; however, differences were not statistically significant. This may indicate that cardiac alterations linked to sickle cell disease worsen with age, and this might need further wide-scale studies [52].The tissue Doppler echocardiographic-derived left ventricular MPI played an essential role in the present study in reflecting the process of myocardial fibrosis through its relation to the extracellular volume and serum level of galectin-3.

Our study has some limitations. First, not all controls were subjected to cardiac MRI and we did not administer contrast to the controls who did have cardiac MRI as there is no ethical justification in our institute. This was a single-center study and studies with larger cohorts and longer follow-up are recommended. Children under the age of eight years were not included in our study as younger children usually require anesthesia for MRI scans. Finally, not investigating patients during their vaso-occlusive crises may be considered a limitation.

## Conclusion

Early detection of interstitial myocardial fibrosis and the determination of extracellular volume in pediatric patients with sickle cell disease is feasible with the use of T1 mapping. This observed fibrosis was generally diffuse rather than focal and was not explained by myocardial iron overload, which was normal in our patient population. An ischemia–reperfusion injury hypothesis would potentially be supported by our results. T1 values and extracellular volume abnormalities correlate well with galectin-3 and tissue Doppler echocardiographic-derived left ventricular MPIm, and these tools are recommended for the detection of early subclinical myocardial abnormalities in children with sickle cell disease.

### Supplementary information

Below is the link to the electronic supplementary material.

Supplementary file1 (DOCX 30 KB)

## Data Availability

Data will be available on reasonable request.
