# Supplementary material for: Evaluation of cardiac fibrosis and subclinical cardiac changes in children with sickle cell disease using magnetic resonance imaging, echocardiography, and serum galectin-3
Source: Pediatr Radiol. 2023 Sep 16;53(12):2515–27. doi: 10.1007/s00247-023-05750-2 (PMC10635955; doi:10.1007/s00247-023-05750-2)
Supplement: Supplementary file 1 — Supplementary file1 (DOCX 30 KB) [file 247_2023_5750_MOESM1_ESM.docx]

**Methodology of Cardiac MRI:**

Cardiac MRI was conducted on 1.5 Tesla MRI scanner (Philips ingenia MR systems, the Netherlands).

- **Image acquisition:** All images were ECG gated using retrospective gating with 25 images reconstructed per cardiac cycle. The images were acquired during breath hold (in the end expiratory phase) in all patients except those who were unable to hold their breath where free breathing was the alternative.
- **Sequences:**
- Scout**:** To delineate the acquisition's field of view (FOV) through obtaining images of the heart in the three orthogonal orientations for the planning of the subsequent images.
- Cine images: Images were acquired using bright blood steady state free precession (SSFP) sequence to assess anatomy, ventricular and valvular functions. Normal values will be based on the values reported by J.P.G. van der Ven et al[[1](#_ENREF_1)] and [Daniëlle Robbers-Visser](https://pubmed.ncbi.nlm.nih.gov/?term=Robbers-Visser+D&cauthor_id=19243036) et al [[2](#_ENREF_2)].
- **T2* sequence** for measuring myocardial T2* value, a single short-axis midventricular slice and cutting through the right hepatic lobe was acquired using a single breath-hold ECG-gated multi-echo technique (TR: 710 ms, slice thickness: 10 mm, flip angle: 20°, FOV: 400 × 300 mm, matrix: 256 × 96 mm, bandwidth: 810 Hz/Px). This T2* sequence generated a series of twelve images with TEs of 1-12 ms and echo spacing of 2 ms, then region of interest (ROI) was drawn within the interventricular septum and at the right hepatic lobe (excluding major vascular branches) in each image to measure signal intensity. Myocardial and hepatic T2* decay was calculated by plotting the mean signal intensity of each image against TE for each image. The T2*value was then calculated from the resulting exponential decay curve. In heavily-iron loaded tissues, signal decay occurred rapidly with noise dominating the signal at later echo times, to minimize it truncation was done according to He,T et al [[3](#_ENREF_3)]. Myocardial iron concentration (MIC) and liver iron concentration (LIC) were calculated. According to cardiac and hepatic T2* values, iron overload risk stratification in patients will be done according to [Wafaa Abdallah](https://www.semanticscholar.org/author/Wafaa-Abdallah/49072858) et al [[4](#_ENREF_4)], Carpenter, J. P et al [[5](#_ENREF_5)] and Triadyaksa, P et al [[6](#_ENREF_6)].
- **T1 mapping:** T1 quantification was performed with motion-corrected (MOCO) modified Look-Locker Inversion recovery (MOLLI) sequence acquired pre and 10 minutes following bolus contrast administration with variable inversion preparation times. Slice thickness 10 mm, TR 2.8 ms, TE 1.3 ms, Flip angle =35º, voxel size=1.9×1.9 x 10mm, parallel imaging=1. The accuracy of the T1-relaxation measurements of the scanner was determined at the beginning of the study using a standard, commercially available relaxation phantom at room temperature. To ensure accurate T1-measurements and exclusion of blood T1 pixels that may affect the accuracy of the measurements, the regions of interest (ROI) were drawn very conservatively to exclude blood pool, papillary muscles, chordae, and trabeculations. Basal, mid and apical short-axis images were used, and the mean of the LV 17 segments measurements was taken as a representative of the LV native, post contrast T1 times and ECV. The endocardial & epicardial borders as well as ROI within the blood pool was drawn in pre and post contrast T1 map images. Evaluation of Myocardial ECV: images were automatically assessed taking into consideration the hematocrit value obtained on the same day of the study.

ECV then was calculated using the following equation [[7](#_ENREF_7)]

$$\boldsymbol{ECV=(1-hematocrit)}\frac{\left( \boldsymbol{1\div T}\boldsymbol{1 myo post} \right)\boldsymbol{-(1\div T}\boldsymbol{1 myo pre)}}{\left( \boldsymbol{1\div T}\boldsymbol{1 blood post} \right)\boldsymbol{- (1\div T}\boldsymbol{1 blood pre)}}$$

Native (pre contrast) T1 results were compared to normal values from 15 healthy individuals (mean age 15.2 years) obtained using the same scanner. The ECV values are not calculated in controls to avoid unnecessary contrast administration. Hence, the results will be compared to the cut off value 20.8% ± 2.4 using Pagano JJ [[8](#_ENREF_8)] results who studied the same age group to our study.

- **Late gadolinium enhancement:**

Phase sensitive inversion recovery (PSIR) fast gradient echo sequences were used for totally nulling the normal myocardium and detection of hyperenhancement (fibrosis) in the abnormal myocardium. Typical scan parameters were repetition time=6.1 ms, echo time=3 ms, matrix= 188x153, 8 mm slice thickness, flip angle= 25°.Visual assessment of presence or absence of myocardial fibrosis and its distribution if any (sub-endocardial, sub-epicardial or transmural)

- **Postprocessing:**

All the post processing was done by two experienced observers blind to the clinical status of the patient on dedicated software (Phillips intelliSpace Portal version 11.0).

1. van der Ven JPG, Sadighy Z, Valsangiacomo Buechel ER, Sarikouch S, Robbers-Visser D, Kellenberger CJ, Kaiser T, Beerbaum P, Boersma E, Helbing WA (2020) Multicentre reference values for cardiac magnetic resonance imaging derived ventricular size and function for children aged 0-18 years. European heart journal Cardiovascular Imaging 21:102-113

2. Robbers-Visser D, Boersma E, Helbing WA (2009) Normal biventricular function, volumes, and mass in children aged 8 to 17 years. Journal of magnetic resonance imaging : JMRI 29:552-559

3. He T, Gatehouse PD, Smith GC, Mohiaddin RH, Pennell DJ, Firmin DN (2008) Myocardial T2* measurements in iron-overloaded thalassemia: An in vivo study to investigate optimal methods of quantification. Magnetic resonance in medicine 60:1082-1089

4. Abdallah W, Ibrahim A, Eissa HA, Abdel-Rahman AS (2021) ROLE OF CARDIOVASCULAR MAGNETIC RESONANCE IMAGING IN ASSESSMENT OF MYOCARDIAL IRON OVERLOAD IN THALASSEMIA PATIENTS. Ain Shams Medical Journal

5. Carpenter JP, He T, Kirk P, Roughton M, Anderson LJ, de Noronha SV, Baksi AJ, Sheppard MN, Porter JB, Walker JM, Wood JC, Forni G, Catani G, Matta G, Fucharoen S, Fleming A, House M, Black G, Firmin DN, St Pierre TG, Pennell DJ (2014) Calibration of myocardial T2 and T1 against iron concentration. Journal of cardiovascular magnetic resonance : official journal of the Society for Cardiovascular Magnetic Resonance 16:62

6. Triadyaksa P, Oudkerk M, Sijens PE (2020) Cardiac T(2) * mapping: Techniques and clinical applications. Journal of magnetic resonance imaging : JMRI 52:1340-1351

7. Gai ND, Sandfort V, Liu S, Lima JAC, Bluemke DA (2016) Dose correction for post-contrast T1 mapping of the heart: the MESA study. The international journal of cardiovascular imaging 32:271-279

8. Pagano JJ, Yim D, Lam CZ, Yoo SJ, Seed M, Grosse-Wortmann L (2020) Normative Data for Myocardial Native T1 and Extracellular Volume Fraction in Children. Radiology Cardiothoracic imaging 2:e190234
